# They Show Progress

**Published:** 1916-05

**Authors:** 


### They Show Progress.

Have you ever noticed that the physicians who take a number of good medical journals and who read them closely are the physicians who stand highest in the profession. They are the most prominent in medical conventions, prepare and read the best papers, and are looked upon as leaders. The reason is plain to see; they are students; they keep right up with the best thought of the profession and consequently have the best practice. Too many physicians think that medical diplomas and a few standard medicines to prescribe on all occasions are all that they need. Physicians who study medical journals are not likely to "mossify."

